# tRNA-derived small RNAs in human cancers: roles, mechanisms, and clinical application

**DOI:** 10.1186/s12943-024-01992-2

**Published:** 2024-04-15

**Authors:** Manli Zhou, Xiaoyun He, Jing Zhang, Cheng Mei, Baiyun Zhong, Chunlin Ou

**Affiliations:** 1 Department of Pathology, Xiangya Hospital, Central South University, Changsha, Hunan 410008 China; 2 Department of Clinical Laboratory, Xiangya Hospital, Central South University, Changsha, Hunan 410008 China; 3 Departments of Ultrasound Imaging, Xiangya Hospital, Central South University, Changsha, Hunan 410008 China; 4 Department of Blood Transfusion, Xiangya Hospital, Clinical Transfusion Research Center, Central South University, Changsha, Hunan 410008 China; 5 National Clinical Research Center for Geriatric Disorders, Xiangya Hospital, Central South University, Changsha, Hunan 410008 China

**Keywords:** tRNA-derived small RNAs, Extracellular vesicles, Metabolic reprogramming, Tumor microenvironment, Liquid biopsy, Therapeutic target

## Abstract

Transfer RNA (tRNA)-derived small RNAs (tsRNAs) are a new type of non-coding RNAs (ncRNAs) produced by the specific cleavage of precursor or mature tRNAs. tsRNAs are involved in various basic biological processes such as epigenetic, transcriptional, post-transcriptional, and translation regulation, thereby affecting the occurrence and development of various human diseases, including cancers. Recent studies have shown that tsRNAs play an important role in tumorigenesis by regulating biological behaviors such as malignant proliferation, invasion and metastasis, angiogenesis, immune response, tumor resistance, and tumor metabolism reprogramming. These may be new potential targets for tumor treatment. Furthermore, tsRNAs can exist abundantly and stably in various bodily fluids (e.g., blood, serum, and urine) in the form of free or encapsulated extracellular vesicles, thereby affecting intercellular communication in the tumor microenvironment (TME). Meanwhile, their abnormal expression is closely related to the clinicopathological features of tumor patients, such as tumor staging, lymph node metastasis, and poor prognosis of tumor patients; thus, tsRNAs can be served as a novel type of liquid biopsy biomarker. This review summarizes the discovery, production, and expression of tsRNAs and analyzes their molecular mechanisms in tumor development and potential applications in tumor therapy, which may provide new strategies for early diagnosis and targeted therapy of tumors.

## Introduction

Tumors, a malignant disease characterized by the uncontrolled growth and replication of cancerous cells, infiltration into nearby tissues, and subsequent organ failure, pose severe morbidity and mortality risks [1]. Despite considerable progress in comprehending and treatment of tumors in recent years, frequent tumor recurrence and metastasis result in high morbidity and mortality [2, 3]. Significant challenges in tumor therapy include the incomplete elucidation of tumorigenesis mechanisms and the lack of clinical trials for tumor-specific therapies. These limitations impede drug development in this field [4–6]. Early screening and identification are crucial clinical strategies to improve the long-term survival of patients with tumors [7]. Understanding tumorigenesis mechanisms and exploring potential diagnostic markers and therapeutic targets related to tumors are pivotal for advancing tumor-targeted therapies and devising more effective treatment strategies.

The post-genomic era has contributed significantly to understanding the genome, with a growing research focus on non-coding RNAs (ncRNAs) [8, 9]. ncRNAs encompass RNA molecules transcribed from the genome that lack protein-coding capabilities. They are classified based on their sizes into two types: long non-coding RNAs (lncRNAs, > 200 nt) and small non-coding RNAs (sncRNAs, < 200 nt) [10, 11]. sncRNAs comprise diverse categories, including microRNAs (miRNAs, 18–25 nt), PIWI-interacting RNAs (piRNAs, 18–30 nt), and tRNA-derived small RNAs (tsRNAs, 15–40 nt) [12–14]. tsRNAs, discovered in recent years, are an emerging class of sncRNAs with lengths ranging from 15 to 40 nt [15]. Notably, tsRNAs are not byproducts produced by random degradation of transfer RNAs (tRNAs), but rather fragments produced by the cleavage of tRNA precursors (pre-tRNAs) or mature tRNAs at specific sites [16]. Depending on the cleavage site, tsRNAs can be stratified into different types, such as tRNA-derived fragments (tRFs) and tRNA halves (tiRNAs) [17]. Moreover, tRNAs can fulfill diverse biological functions through various mechanisms. These molecules can interact with mRNAs or proteins within the cell, participating in various cellular functions like signaling, gene expression regulation, cell cycle modulation, epigenetic control, and modification of chromatin [18–21]. Extracellular vesicles (EVs), a class of vesicle bodies with a double-layered phospholipid membrane structure, are released by almost all cells into the external cellular environment. They are mainly divided into three subpopulations according to their diameter: exosomes (30–150 nm in diameter), microvesicles (100–1000 nm in diameter), and apoptotic bodies (100–5000 nm in diameter) [22–26]. Increasing evidence indicates that tsRNAs can be encapsulated within EVs and transported to the periplasm of target cells and extracellularly through the in vivo microenvironment, where they play an important role in “cell-cell” communication [27–29]. Therefore, mutation, modification, or dysregulation of tsRNAs can trigger the onset of various human diseases. These include Parkinson’s disease, Alzheimer’s disease, diabetic retinopathy, mitochondrial encephalomyopathy, lactic acidosis, and others. Additionally, these abnormalities in tsRNAs are associated with different cancers, encompassing non-small cell lung cancer (NSCLC) and gastric cancer (GC) [30–34]. Furthermore, tsRNAs actively participate in the physiopathological mechanisms of multiple solid tumors and hematological malignancies. They exhibit distinct expression patterns between patients with cancer and normal controls. These discoveries present extensive opportunities for identifying biomarkers based on tsRNAs and potential therapeutic targets [35–38].

This review encapsulates the discovery, generation, expression, and role of tsRNAs. In addition, it delves into the molecular mechanisms via which tsRNAs operate in tumorigenesis and elucidates their potential applications in tumor therapy. Additionally, this study explores the potential of tsRNAs as markers in liquid biopsies for early tumor detection, monitoring disease progression, and assessing prognosis. Taken together, the outcomes of this study offer a fresh perspective on the potential of tsRNAs as novel biomarkers for diagnosing tumors and as targets for therapeutic interventions.

## Discovery, biogenesis and regulatory mechanism of tsRNAs

### Discovery and identification of tsRNAs

**Fig. 1:**
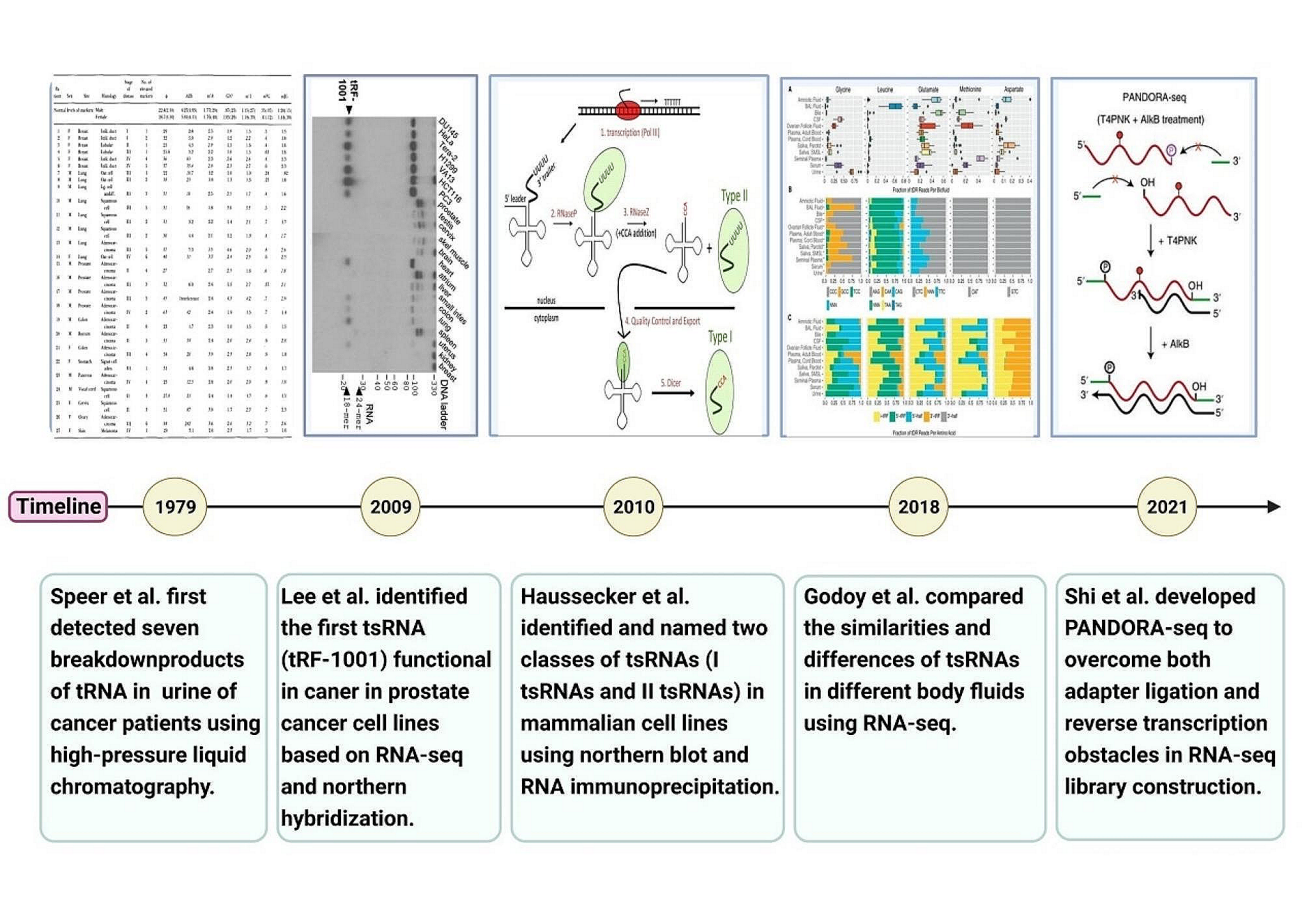
Timeline of key discoveries of tsRNAs

In 1979, Speer et al. [39] first detected seven types of tRNA decomposition products in the urine of 27 cancer patients with 13 different malignancies using high-pressure liquid chromatography. They found that the levels of these decomposition products may correspond to the severity of the disease, although the precise underlying processes remain unclear. In 2009, Lee et al. [40] identified the first tsRNA (tRF-1001) acting in cancer in prostate cancer (PC) cell lines via RNA-seq and Northern hybridization. The acquired data demonstrated the significant role of the tsRNAs in cell proliferation, indicating that these are not random byproducts produced by tRNA degradation but rather a novel class of small RNAs with specific biological functions. Subsequently, in 2010, Haussecker et al. [41] identified two types of 5′-phosphate, 3′-hydroxylated human tsRNAs in HEK-293 and HCT116 human cell lines via Northern blot and RNA immunoprecipitation. These tsRNAs were named “Type I tsRNAs” and “Type II tsRNAs”. They further studied the mechanism and found that the expression level of tsRNAs is related to the silencing activity of miRNAs and small interfering RNAs (siRNAs). In 2018, Godoy et al. [42] used RNA-seq to compare the similarities and differences of tsRNAs in 12 different body fluids, including plasma, serum, urine, amniotic fluid, bronchoalveolar lavage fluid, bile, cerebrospinal fluid, et al. The analysis data implied that tsRNAs may serve as a novel type of biomarker. Subsequently, in 2021, Shi et al. [43] developed PANDORA-seq based on RNA-seq, improving the adapter ligation and reverse transcription issues during RNA-seq library construction. This technology was successful in discovering previously undetected tsRNAs in mouse and human tissues and cells, revealing the change of tsRNAs during somatic cell reprogramming to induced pluripotent stem cells (iPSCs). The findings suggest that tsRNAs may be crucially involved in the differentiation process of embryonic stem cells (ESCs) (Fig. 1).

### Biogenesis of tsRNAs

tRNAs are a type of ncRNAs that participate in protein synthesis and other cellular activities by transporting specific amino acids [44, 45]. tRNAs are arranged in a cloverleaf-like secondary structure, consisting of four arms (D arm, anticodon arm, TψC arm, acceptor stem) and four loops (D loop, TψC loop, anticodon loop, variable loop) [46]. In eukaryotes, tRNA genes are catalyzed by RNA polymerase III (Pol III) in the cell nucleus to generate pre-tRNAs through three stages: initiation, elongation, and termination. Subsequently, pre-tRNAs go through a sequence of processing and modification processes to become active mature tRNAs [47–49]. Different endonucleases such as angiogenin (ANG), RNase Z, Dicer, etc., can cleave specific sites of pre-tRNAs or mature tRNAs, thereby producing different categories of tsRNAs [50, 51]. Based on the cleavage sites identified in tRNAs, tsRNAs can be broadly categorized into two groups: tiRNAs and tRFs [52].

**Fig. 2:**
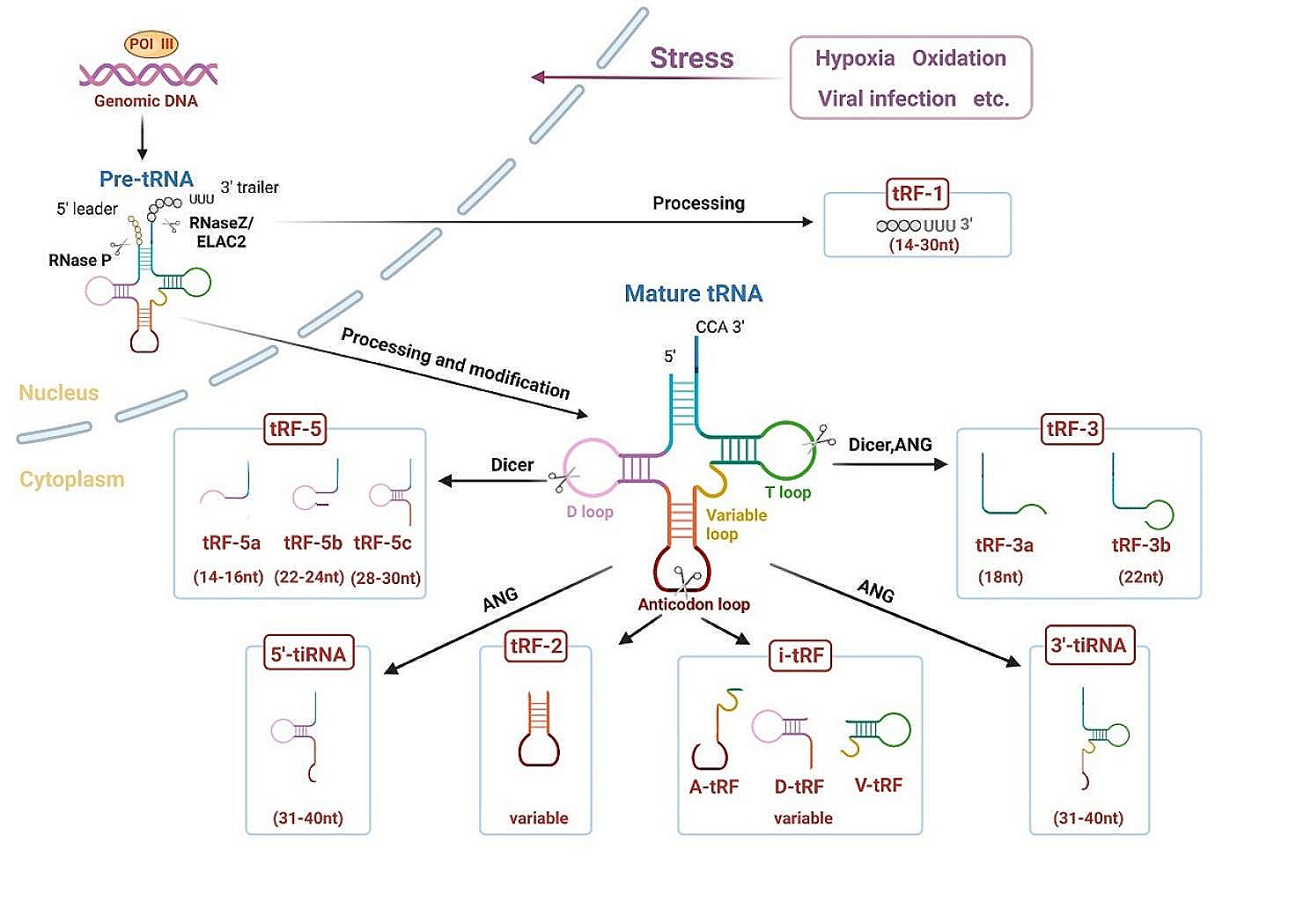
Biogenesis and classification of tsRNAs. Pre-tRNAs are first transcribed from tRNA genes via RNA polymerase III in the nucleus, which undergo a series of processing and modification processes (e.g., m5C, m7G, and Ψ) and are converted into active mature tRNAs. tsRNAs can be broadly categorized into tiRNAs and tRFs as per the cleavage sites within tRNAs. Under stress conditions, tiRNAs are produced through ANG cleavage of the anticodon loops found in mature tRNAs, encompassing 5’-tiRNAs and 3’-tiRNAs. tRFs emerge from pre-tRNAs or fully mature tRNAs and are categorized per the initial cleavage sites into tRF-1, tRF-3, tRF-5, tRF-2, and i-tRF. Specifically, tRF-1 arises from the cleavage of pre-tRNA facilitated by endonuclease (RNase Z/ELAC2). tRF-3 can arise from the cleavage of the TΨC loop within mature tRNAs, a process facilitated by enzymes such as Dicer and ANG. This category is then subdivided into tRF-3a and tRF-3b. Additionally, tRF-5 is produced via cleavage of the D-loop, D stem, or 5’ half of the anticodon stem of mature tRNAs by Dicer and is classified as tRF-5a, tRF-5b, and tRF-5c. tRF-2 is produced by anticodon loops under hypoxic stress stimulation. i-tRF is mainly generated from the internal regions of mature tRNAs and is classified as A-tRF, D-tRF, and V-tRF.

tiRNAs are generated by ANG cutting the anticodon loop of the mature tRNAs under stress conditions, for example, hypoxia, UV irradiation, oxidation, amino acid deficiency, heat shock, starvation, viral infection, etc. The process results in fragments of 31–40 nt in length [37, 53]. Depending on whether the 5’ or 3’ sequence of the anticodon loop cleavage site is included, tiRNAs can be classified into 5’-tiRNA (31–40 nt) and 3’-tiRNA (31–40 nt) [54, 55]. tRFs, ranging in length from 14 to 30 nucleotides, can emerge from either pre-tRNAs or mature tRNAs. These fragments are categorized into distinct types: tRF-1, tRF-3, tRF-5, tRF-2, and i-tRF, based on their specific sites of origin within these molecules [56]. Among them, tRF-1 (14–30 nt) is generated by RNase Z/ELAC2 catalyzing the cleavage of the 3’ end of pre-tRNAs and has a poly U characteristic, thus also known as 3’UtRF [57]. tRF-3 is produced by Dicer and ANG cleaving the TψC loop of mature tRNAs and can be further stratified into tRF-3a (18 nt) and tRF-3b (22 nt) based on its length [32]. tRF-5 can be generated by Dicer cleaving the D-loop, D stem, or 5’ half of the anticodon stem of mature tRNAs. According to its different cleavage sites, tRF-5 can be categorized into three specific subtypes: (1) tRF-5a (14–16 nt); (2) tRF-5b (22–24 nt); (3) tRF-5c (28–30 nt) [58]. tRF-2 and i-tRF are recently discovered atypical tsRNAs derived from tRNAs, produced by nuclear-encoded and mitochondrial-encoded tRNAs, present in different tissues and individuals [56, 59]. Specifically, tRF-2 is generated under hypoxic stress from the anticodon loop, while i-tRF mainly comes from the internal region of mature tRNAs. In addition, i-tRF can be categorized into A-tRF, D-tRF, and V-tRF based on different cleavage start sites [60] (Fig. 2). However, the detailed biological occurrence and mechanism of these two tsRNAs remain unclear.

Although the potential mechanisms regulating the biogenesis of tRFs remain unclear, increasing evidence suggests that tRNA modifications, such as 5-methylcytosine (m5C), N7-methylguanosine (m7G), and pseudouridine (Ψ), play significant roles in tRNA cleavage and fragment generation [57]. On one hand, tRNA modifications can increase the stability of tRNA structure, protecting them from being cleaved by nucleases to produce tsRNAs. For instance, Zhu et al. [61] reported that METTL1-mediated m7G modification regulates the tRNAs stability under sublethal heat stress and protects tRNAs from being cleaved into tsRNAs in hepatocellular carcinoma (HCC) cells. They also discovered that METTL1-mediated m7G tRNA modification enhances malignant expression in HCC cells after sublethal heat stress in various experimental models, including patient-derived HCC xenograft (HCC-PDX) mouse models, patient HCC tissues, sublethal-heat-treated HCC cell line model, and HCC patient-derived organoids. Similar results were found in PC studies. Researchers discovered that METTL1-mediated m7G tRNA modification can protect tRNAs from stress-induced cleavage and the generation of tRF-5 fragments. Conversely, the loss of METTL1-mediated tRNA methylation disrupts tRNA processing, leading to autophagy inhibition, decreased cellular viability, DNA damage, and protein homeostasis imbalance [62]. In addition, Chen et al. [63] observed that highly enriched tsRNAs in mature sperm can serve as carriers of epigenetic information, transmitting the phenotype of high-fat-induced metabolic disorder from parents to offspring. Subsequent investigations revealed that this may be related to the spectrum of sperm sncRNAs and associated RNA modifications; the loss of RNA methyltransferase DNMT2 leads to hypomethylation of m5C, promoting tRNA fragmentation, altering the biological properties of tsRNAs and thus affecting the phenotype of the offspring [64]. On the other hand, some RNA modifications can promote the biogenesis of tsRNAs. For example, researcher has revealed that the stem cell-enriched Ψ “writer” PUS7 can activate the biogenesis of tRFs, further forming Ψ-bearing tRF-5 [65].

In summary, tsRNAs are not simply degradation byproducts. Different types of tsRNAs have different biogenesis processes and may also have different biological functions.

### Regulatory mechanism of tsRNAs

**Fig. 3:**
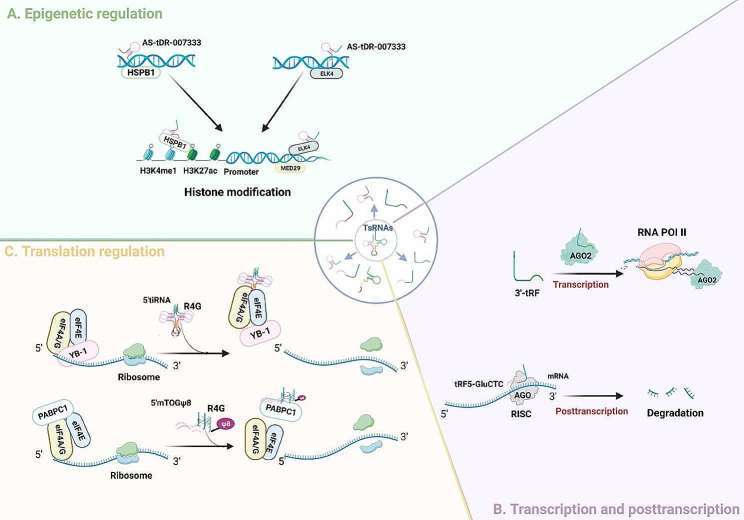
Regulatory mechanism and biological function of tsRNAs. **A** Epigenetic regulation. AS-tDR-007333 can interact with HSPB1-MED29 and ELK4-MED29 for the epigenetic regulation of target genes. **B** Transcriptional and post transcriptional horizontal regulation. Dicer dependent 3′-tRF inhibits the expression of related genes by binding to the AGO2 protein and introns of newly generated RNA in the nucleus; tRF5-GluCTC can interact with AGO protein to induce the formation of RISC, thereby inducing the post transcriptional gene silencing. **C** Translation regulation. Assembly of 5′-tiRNA into R4G displaces translation initiation complex eIF4A/G/E for translation inhibition; 5’mTOGΨ8 can also form an R4G structure, replacing PABPC1 from the translation initiation complex of mRNA and inhibiting mRNA translation

Although the functions of tsRNAs have not yet been elucidated, an increasing number of studies have shown that tsRNAs play their biological functions in multiple regulatory mechanism, including epigenetic regulation, transcriptional or post-transcriptional regulation, and translation regulation [66] (Fig. 3).

#### Epigenetic regulation

Epigenetics is the study of reversible and heritable alterations in gene function that occur without any changes to the underlying DNA sequence. Abnormalities in the epigenetic regulation of gene function are intricately linked to the onset and advancement of diverse diseases, including cancer [67, 68]. The main research areas in epigenetics include DNA methylation, histone modifications, chromatin remodeling, and ncRNA regulation [69]. Multiple studies indicate that tsRNAs may regulate different epigenetic processes.

tsRNAs can regulate gene expression without altering DNA sequences by controlling histone modifications and chromatin accessibility [70]. The recent discovery of super enhancers (SEs) has provided new insights into the epigenetic regulatory mechanisms of tumorigenesis and cancer development. SEs are a class of cis-regulatory elements with super-enhanced transcriptional activation characteristics, showing higher densities of transcriptionally active chromatin modifications (such as H3K27ac, H3K4me1, etc.) compared to typical enhancers. They are involved in regulatory functions in important biological processes such as cancer occurrence, metastasis, and immune response [71–73]. In a recent investigation, it was revealed that tsRNAs are involved in the regulation of H3K27ac and H3K4me1 modifications, thereby promoting the occurrence and progression of NSCLC. Researchers found that the highly expressed AS-tDR-007333 in NSCLC cells enhances proliferation and migration by activating the HSPB1-MED29 and ELK4-MED29 axis. Chromatin immunoprecipitation (ChIP)-qPCR assays highlighted that downregulating HSPB1 decreases the expression of histone modifications (H3K27ac, H3K4me1) at the MED29 promoter, suggesting that AS-tDR-007333 may be a promising potential therapeutic target for NSCLC [74]. In addition, researchers found that 5’tRF-Gly-GCC (tRF-GG) functions as an endogenous retroelement inhibitor and regulates the chromatin formation of MERVL-associated genes. Mechanistically, MERVL can be wrapped and repressed by heterochromatin, and tRF-GG controls histone expression by controlling the production of U7 ncRNA. This, in turn, impacts the expression of MERVL-associated genes and alters chromatin accessibility within heterochromatin [75]. U7 ncRNA can recognize histone pre-messenger RNA and participates in its 3’ end processing [76].

#### Transcriptional and post-transcriptional regulation

Gene expression regulation determines the characteristics and functions of cells, playing a crucial role in the development, aging, and disease processes, as gene expression is precisely controlled at the transcriptional level [77]. Increasing studies shows that tsRNAs can regulate the transcription/post-transcription levels of specific genes through various mechanisms.

tsRNAs can bind with the endonuclease Dicer and load into AGO protein, inducing the formation of the RNA-induced silencing complex (RISC), thereby regulating gene expression at the transcription/post-transcription level [36]. Recently, a novel mechanism of Dicer-dependent tRF-mediated RNA silencing has been reported. Dicer-dependent 18–22 nt 3’-tRFs interact with AGO2 protein and introns in the nucleus, inhibiting the expression of many genes associated with various cancers [78]. Additionally, Choi et al. [79] demonstrated the important role of the 5’ end tRNA-derived RNA fragment (tRF5-GluCTC) from mature tRNA in gene regulation. The tRF5-GluCTC induced by respiratory syncytial virus (RSV) is critical at the post-transcriptional level for RSV replication and gene silencing. Mechanistically, tRF5-GluCTC, by binding with AGO4 protein, forms RISC and exerts gene silencing function. Target identification based on tRF5-GluCTC may pave the way for new antiviral strategies. In another study, Hasler et al. [80] revealed that pre-tRNA-IIe can mediate the generation of functional miRNA (has-miR-1983). pre-tRNA-IIe can partially escape tRNA stabilization mediated by the lupus autoantigen La through base-pairing of the 5’ and 3’. La is an ssRNA-binding protein that stabilizes Pol III and supports RNA folding, and is also involved in the miRNA synthesis pathway. Furthermore, tsRNAs are involved in regulating m6A modifications and influencing related gene expressions. Overexpression of tRF-22 has been found to alleviate choroidal vasculopathy, delaying the progression of myopia. Mechanistically, tRF-22 directly binds to the 3’ UTR region of METTL3 mRNA, acting as a miRNA-like molecule to downregulate METTL3 expression and regulate its m6A methylation activity, affecting the Axin1 and Arid1b expression levels. Thus, targeting the tRF-22/METTL3/Axin1/Arid1b axis in the choroid is crucial for the treatment and prevention of myopic pathology [81, 82]. Some recent studies have shown that tsRNAs are also involved in regulating ferroptosis, a unique iron-dependent lipid peroxidation-driven cell death mechanism, which contributes to the onset of diverse diseases and has become a major focus of current medical research [83]. For instance, ferroptosis of pancreatic acinar cells promotes the exacerbation of acute pancreatitis (AP). Researchers found that tRF36 is significantly upregulated in AP and promotes the progression of AP by regulating ferroptosis. In vivo and in vitro experiments show that tRF36 promotes ferroptosis of pancreatic acinar cells by binding with insulin-like growth factor 2 mRNA binding protein 3 (IGF2BP3). Conversely, reducing tRF36 decreases cell death in pancreatic acinar cells. The discovery of tRF36 may serve as a potential biomarker for AP diagnosis [84]. In addition, tRFs can regulate the reverse transcription of retrotransposons. Yeung et al. [85] discovered the 18nt tRF derived from tRNALys (PBSncRNA) during high-throughput pyrosequencing of HIV-infected T cells. PBSncRNA is commonly used as a primer for reverse transcription and cDNA synthesis. Mechanistically, PBSncRNA binds with AGO, targeting intracellularly viral genomic sequence, hindering the synthesis of retroviral cDNA and affecting HIV gene replication.

#### Translation regulation

Protein synthesis in eukaryotes is a central and essential biological process involving a series of steps, including transcription of mRNA within the cell nucleus, processing, and export to the cytoplasm for translation by ribosomes [86]. In tumors, dysregulation of translation control is often considered to be a tumor-promoting process aimed at accelerating the production of biomolecules such as proteins [87]. Recent research findings indicate that tsRNAs can regulate protein translation by influencing ribosome biogenesis and the transcription of ribosomal protein genes [88].

Ivanov et al. [89] studied the mechanism of tsRNA suppression of protein translation. They found that 5’-tiRNAAla-binding proteins can assemble into RNA G-quadruplexes (R4G) and displace the translation initiation complex eIF4A/G/E together with Y-box-binding protein 1 (YB-1), inhibiting mRNA translation initiation. YB-1, belonging to the cold shock protein family, is a crucial regulator of transcription and translation, exerting significant influence on tumor invasion and metastasis. Guzzi et al. [65] concluded similar results in their study on the regulatory mechanism of ESCs protein production. They discovered that the 18-nt TOG-5’tsRNA with a pseudouridine modification at position 8 (Ψ8) can inhibit translation. Mechanistically, 5’mTOGΨ8 can also form an R4G structure, which, upon binding with poly(A)-binding protein cytoplasmic 1 (PABPC1), displaces PABPC1 from the mRNA, leading to translation inhibition. Conversely, when lacking pseudouridine modification, this RNA does not possess the translation inhibition function. Furthermore, Couvillion et al. [90] demonstrated that tRF-3 binding to AGO/PIWI family member Twi12 can regulate ribosome biogenesis. Twi12 can promote exonuclease Xrn2 and Tan1 to form TXT, a nuclear complex with 5’-monophosphate-dependent exonuclease activity. Conversely, depletion of Twi12 or Xrn2 leads to defects in rRNA processing.

### Methods and tools for the study of tsRNAs

With the continuous advancements in next-generation sequencing (NGS) techniques and high-throughput RNA sequencing, an increasing number of tsRNAs have been discovered in different prokaryotes and eukaryotes, making the study of the biogenesis and functions of tsRNAs a hot topic [91]. Currently, multiple databases related to tsRNAs have been developed and applied, aiding in a more comprehensive understanding of tsRNA data sets and biological functions [92–101] (Table 1). For example, tRFdb is the first database dedicated to studying tRF sequences, containing three classes of tRF sequences (tRF-5, -3, or 1-series) from eight species. It offers coordinates and names of potential tRNA gene genomes that may be the source of tRFs. Users can view tRF sequences and compare their abundance differences across multiple experiments (such as normal controls and cancer patients) [92]. MINTbase v2.0 has been updated, building upon the foundation of the first-generation MINTbase. It now encompasses data on 26,531 different human tRFs acquired from 11,719 human datasets. Users can quickly filter tRFs by minimum abundance threshold and organization keywords, thus learn about the maximum abundance of tRFs and related data set information, and the abundance of tRFs in different cancer types [93]. MINTMap contains data sets of tRFs encoded by mitochondria and nuclei, allowing users to quickly query relevant tRFs data based on their structural categories, nucleic acids, sequences, genome positions, and more. In addition to quickly identifying tRFs sequences, MINTMap can also calculate the raw and normalized abundance of tRFs, and identify tRFs sequences that may come from outside the tRNAs space and label them as candidate false positives, which has high sensitivity and specificity [94]. The tDRmapper offers a systematic method for defining and quantifying tDRs. It analyzes data profiles of tDRs from four distinct cell/tissue type categories. This approach aids in the identification of new tDRs and their associated biological functions [95]. Moreover, tRFexplorer visually represents the expression patterns of tRFs in both the NCI-60 cell line and various tumor types from The Cancer Genome Atlas (TCGA). This tool enables the correlation of tRF expression profiles with other ncRNAs (such as miRNAs) and conducts analyses correlating patient survival with various factors. Additionally, tRFexplorer facilitates the prediction of relevant tRFs, even without direct experimental evidence, aiding in exploring potential biological functions associated with tRFs [96].

**Table 1:** TsRNAs-related databases or tools

Database	Functions	Website	Ref.
tRFdb	The first tRFs database, providing genomic coordinates and names of tRNAs from which tRFs sequences are likely derived.	http://genome.bioch.virginia.edu/trfdb/	[92]
MINTbase v2.0	It can quickly filter tRFs by minimum abundance thresholds and tissue keywords, and provides the maximum abundance of tRFs as well as the related data information.	http://cm.jefferson.edu/MINTbase/	[93]
MINTmap	It contains data sets of tRFs encoded by mitochondria and nuclei, allows rapid identification of tRFs sequences, and calculates the raw and normalized abundance of tRFs.	https://github.com/TJU-CMC-Org/MINTmap/	[94]
tDRmapper	It provides a nomenclature system to define and quantify tDRs, which helps to discover new tDRs and explore more relevant biological functions.	https://github.com/sararselitsky/tDRmapper	[95]
tRFexplorer	It can visualize tRFs expression profiles in NCI-60 cell lines and TCGA tumor types, correlating with other ncRNAs expression profiles (e.g., miRNA) for patient survival correlation analysis.	https://trfexplorer.cloud/	[96]
PtRFdb	It provides 5607 unique tRFs sequences from 10 different plant species.	http://www.nipgr.res.in/PtRFdb/	[97]
tRF2Cancer	It quickly identifies tRFs by analyzing deep sequencing data and contains the expression of tRFs in different types of cancer.	http://rna.sysu.edu.cn/tRFfinder/	[98]
OncotRF	It provides a comprehensive view of tRFs dysregulated in cancers as well as their correlation with clinicopathological features.	http://bioinformatics.zju.edu.cn/OncotRF	[99]
tsRBase	It contains 121,942 tsRNAs from 20 organisms and combines the functional information of tsRNAs with their corresponding targets.	http://www.tsrbase.org	[100]
SPORTS1.0	It can analyze tsRNAs of multiple tissues/cell types, and predicts potential RNA modification sites based on nucleotide mismatches within tsRNAs.	https://github.com/junchaoshi/sports1.0	[101]

## Biological function of tsRNAs in tumor progression

**Fig. 4:**
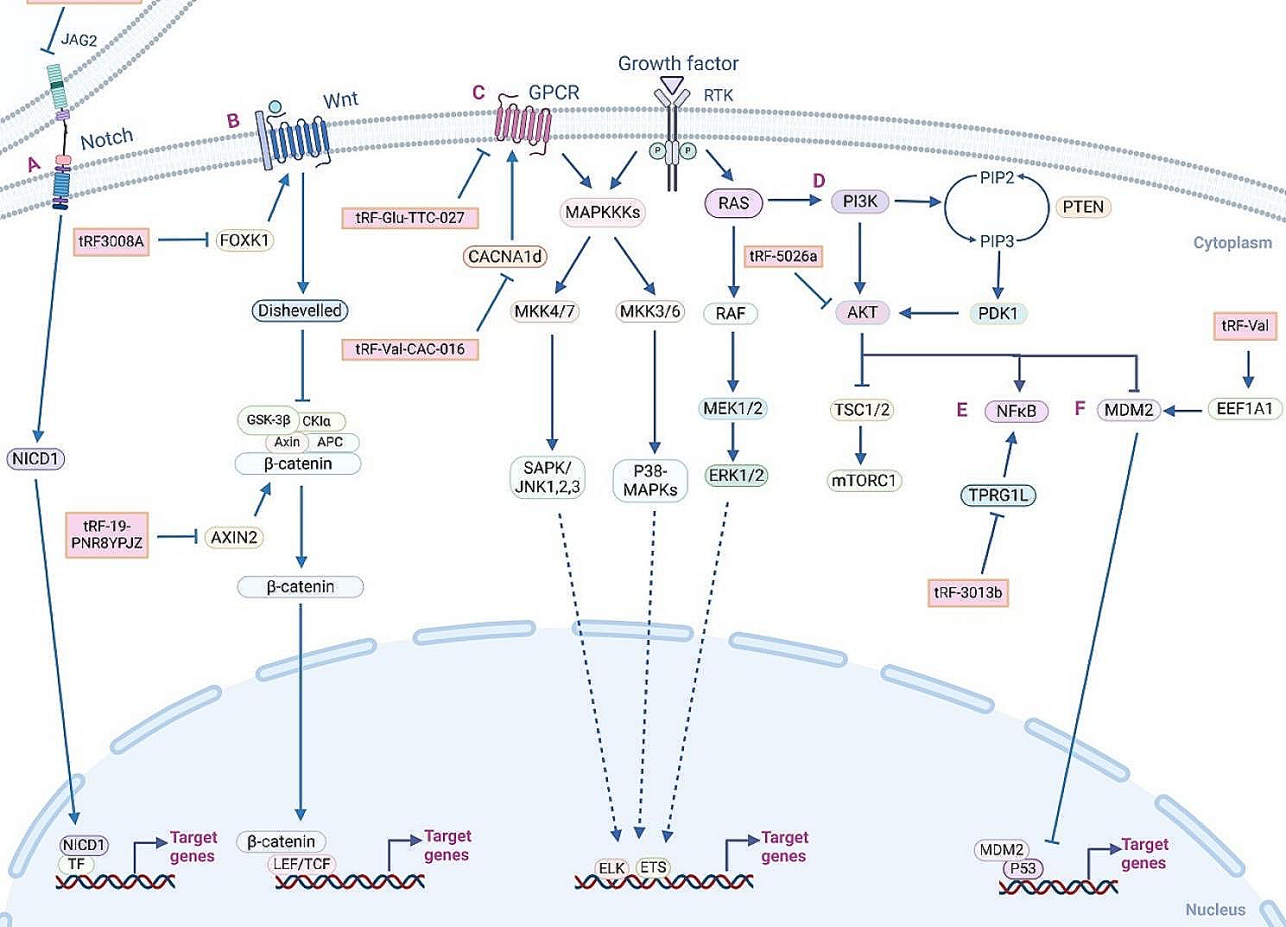
Regulatory mechanism of tsRNAs in cancers. tsRNAs interact with proteins and genes to modulate important signaling cascades within tumor cells, including Notch (e.g., tRF/miR-1280) (**A**), Wnt (e.g., tRF3008A, tRF-19-PNR8YPJZ) (**B**), MAPK (e.g., tRF-Glu-TTC-027, tRF-Val-CAC-016) (**C**), PI3K/AKT (e.g., tRF-5026a) (**D**), NF-κB (e.g., tRF-3013b) (**E**), and MDM2/P53 (e.g., tRF-Val) signaling pathways (**F**)

**Fig. 5:**
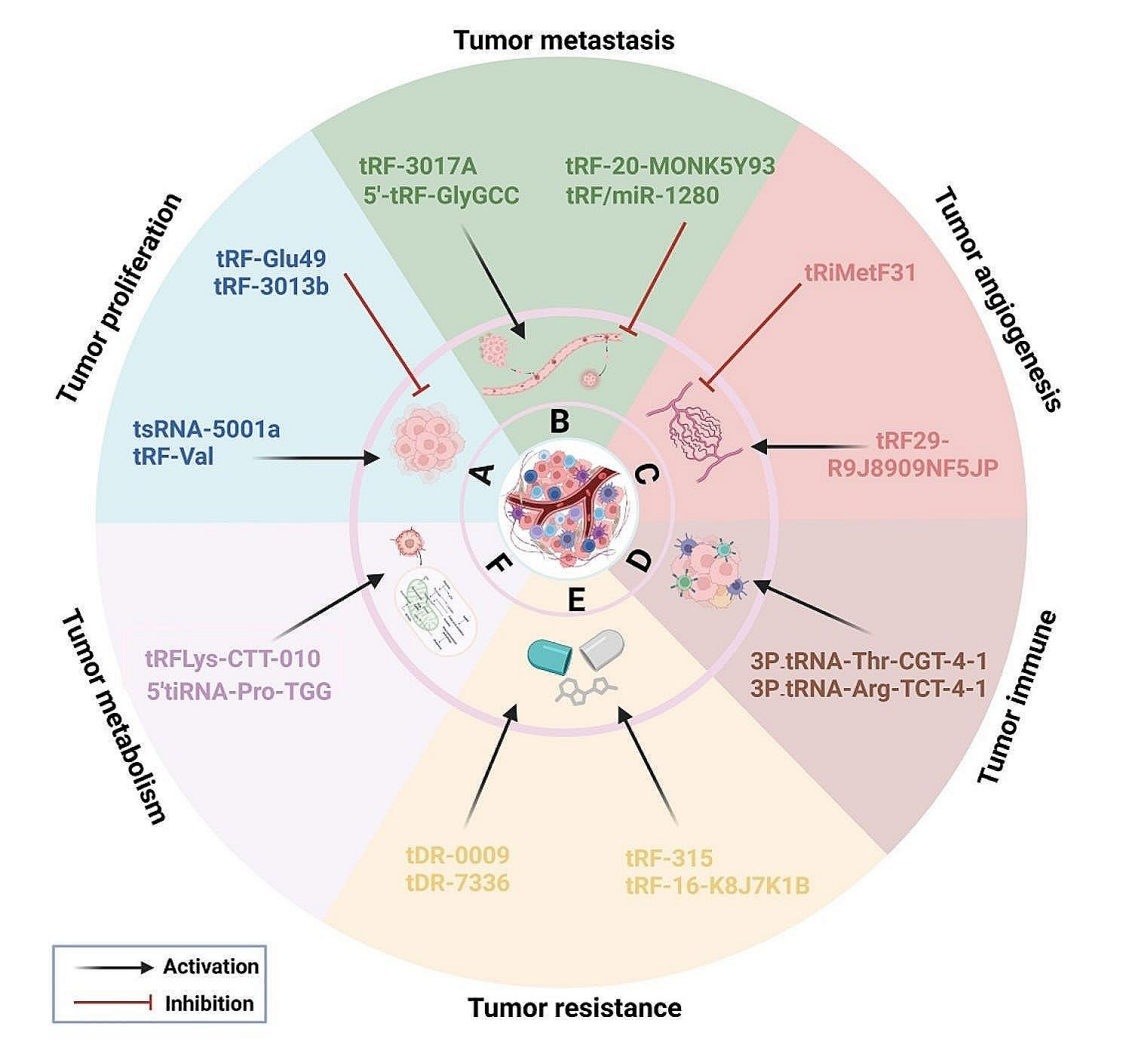
tsRNAs regulate tumorigenesis. **A** tsRNAs (e.g., tsRNA-5001a and tRF-Val) promote tumor proliferation; tsRNAs (e.g., tRF-Glu49 and tRF-3013b) inhibit tumor proliferation. **B** tsRNAs (e.g., tRF-3017 A and 5’-tRF-GlyGCC) promote tumor metastasis; tsRNAs (e.g., tRF-20-M0NK5Y93 and tRF/miR-1280) suppress tumor metastasis. **C** tsRNAs (e.g., tRF29-R9J8909NF5JP) promote tumor angiogenesis; tsRNAs (e.g., tRiMetF31) inhibit tumor angiogenesis. **D** tsRNAs (e.g., 3P_tRNA-Thr-CGT-4-1 and 3P_tRNA-Arg-TCT-4-1) regulate the body’s immune response to NSCLC cells. **E** tsRNAs (e.g., tDR-0009, tDR-7336, tRF-315, and tRF-16-K8J7K1B) promote tumor resistance. **F** tsRNAs (e.g., tRFLys-CTT-010 and 5’tiRNA-Pro-TGG) promote the metabolic reprogramming of tumor cells

Recently, an increasing number of studies have shown that tsRNAs are not only abnormally expressed in cancer cells and tissues, but they can regulate several signaling pathways (Fig. 4), thereby affecting the progression of tumors, including malignant proliferation, invasion and metastasis, angiogenesis, immune response, tumor resistance, and tumor metabolism (Fig. 5).

### tsRNAs and malignant proliferation of tumors

The proliferation of normal cells involves complex signal transduction, and the malignant proliferation of tumor cells is mostly related to the abnormal expression of proliferation-related genes and damage to signaling pathways such as the activation of oncogenes, inhibition of tumor-suppressor genes, and resistance to apoptosis [102–104]. tsRNAs regulate the expression of proliferation-related genes and signaling pathways, possessing pro-tumor or anti-tumor effects.

tsRNAs can act as tumor promoters that stimulate tumor proliferation and play anti-apoptotic roles. Cui et al. [105] demonstrated that the high expression level of tRF-Val, a tRF-3a type, was positively correlated with GC size. Moreover, tRF-Val induces GC cell proliferation and inhibits GC cell apoptosis in vitro. Mechanistically, tRF-Val specifically binds to EEF1A1, mediates its transfer to the nucleus, enhances the interaction between EEF1A1 and the MDM2-p53 complex, and inhibits the downstream molecular pathway of p53, a tumor suppressor protein and transcription factor that acts as “the guardian of the genome” in DNA damage and repair. In another study, using tRF/tiRNA microarray data, Hu et al. [106] revealed that tsRNA-5001a is upregulated in lung adenocarcinoma (LUAD), and overexpression of tsRNA-5001a induces the proliferation of LUAD cells. Further studies showed that tsRNA-5001a targets the growth arrest DNA damage-inducible gene 45 gamma (GADD45G), a tumor suppressor, leading to the downregulation of GADD45G [107]. In a study on NSCLC, Shao et al. [108] found that tRF-Leu-CAG is strongly expressed in NSCLC. tRF-Leu-CAG promotes cell proliferation and cell cycle progression in NSCLC by targeting aurora kinase A (AURKA), a member of the serine/threonine kinase family, and plays an important role in centrosome maturation and chromatin segregation during mitosis. Conversely, tsRNAs can also act as tumor suppressors, and their downregulation may be associated with tumor progression. Zou et al. [109] selected tRF-3013b, which is downregulated in gallbladder cancer (GBC), and found that tRF-3013b inhibits GBC cell proliferation and tumor growth. Flow cytometry analysis showed that tRF-3013b induced G1/S phase arrest in GBC cells, indicating that tRF-3013b targets TPRG1L and inhibits NF-κB, leading to the downregulation of cell cycle regulatory proteins, such as c-Myc and CDK2, thereby inhibiting the proliferation of GBC cells. In addition, in a cervical cancer study, Wang et al. [110] reported that tRF-Glu49 inhibited the proliferation of cervical cancer cells by targeting fibrinogen-like protein-1 (FGL1), an acute inflammatory factor secreted by the liver that is closely related to tumor proliferation and apoptosis [111].

### tsRNAs and tumor metastasis

Tumor metastasis is the process by which malignant tumor cells spread from the primary tumor site to secondary tissues or organs via the circulatory system, where they colonize and grow to form secondary tumors [112, 113]. Tumor invasion and metastasis are complex cascade dynamic processes, including local invasion, intravasation, circulation, extravasation, and colonization of new sites, and are the main causes of cancer-related deaths worldwide [7, 114]. Several mechanisms are involved, such as epithelial-mesenchymal transition (EMT), hypoxia, angiogenesis, and formation of the tumor microenvironment (TME), as well as large changes in cells, including reduced cell adhesion, reconstruction of the cytoskeleton, degradation of the extracellular matrix, and formation of cell protrusions and pseudopodia [115, 116].

EMT is a process in which epithelial cells acquire mesenchymal features consisting of increased migratory and invasive behaviors, which is one of the hallmark events of tumor metastasis that involves the regulation of multiple signaling pathways, such as Notch, Wnt, and TGFβ [117]. Based on in vitro and in vivo experiments, Huang et al. [118] established that tRF/miR-1280 significantly inhibited the metastasis of colorectal cancer (CRC) cells and was notably reduced in patients with CRC. Mechanistically, miR-200b is a member of the miR-200 family and possesses the ability to inhibit EMT and regulate cell division and apoptosis [119]. tRF/miR-1280 expression positively correlates with that of miR-200b, inhibits the Notch signaling pathway by targeting JAG2, and suppresses CRC cell metastasis. Autophagy also plays an important role in tumorigenesis and tumor progression. Chen et al. [120] explored the function and related molecular mechanism of 5’-tRF-GlyGCC in breast cancer (BC) progression. They found that 5’-tRF-GlyGCC is elevated in BC cells, which repressed autophagy and promoted BC cell metastasis. Further studies showed that 5’-tRF-GlyGCC combines with fat mass and obesity-associated protein (FTO) to increase the activity of FTO demethylase, thereby inhibiting autophagy and inducing BC cell invasion and metastasis [121]. These results suggest that 5’-tRF-GlyGCC may be an effective target for BC treatment. Similarly, the activation of oncogenes and the inactivation of tumor suppressors are also closely related to tumor metastasis. Tong et al. [122] established that tRF-3017 A (derived from tRNA-Val-TAC) is significantly increased in GC cells using tRF/tiRNA microarray data and that increased tRF-3017 A levels are associated with elevated lymph node metastasis based on the clinicopathological characteristics of patients with GC. Specifically, tRF-3017 A expression negatively correlates with the expression of the tumor suppressor gene *NELL2*. It exerts miRNA-like effects, induces RISC formation together with AGO2 protein to silence NELL2 expression, and promotes GC cell invasion and metastasis. Moreover, Luan et al. [123] found that tRF-20-M0NK5Y93 binds to specific sites on the oncogene *MALAT1*, a prognostic factor for the metastasis of various cancers, and plays a miRNA-like role by downregulating the expression of MALAT1 and inhibiting CRC cell metastasis by regulating the SMC1A levels. Therefore, tRF-20-M0NK5Y93 may be a potential therapeutic target for CRC.

### tsRNAs and tumor angiogenesis

Tumor growth and metastasis depend on blood vessels for nutrition; hence, continuous angiogenesis is essential for tumor initiation and progression [124]. It involves the regulation of a variety of angiogenic factors and related signaling pathways, such as vascular endothelial growth factor and its receptor, fibroblast growth factor and its receptor, and platelet-derived growth factor and its receptor [125, 126]. In recent years, numerous studies have shown that tsRNAs target angiogenesis-related genes, participate in the regulation of tumor angiogenesis, and are expected to become potential targets for anti-angiogenic therapy.

Wang et al. [127] established that miR-34a-guided tRiMetF31 inhibits BC cell angiogenesis by targeting PFKFB3, a regulator of glycolysis, angiogenesis, and apoptosis [128]. tRiMetF31 abundance is decreased in BC, and knockdown of tRiMetF31 expression restores PFKFB3-driven angiogenesis, suggesting that tRiMetF31 may act as a potential target for the diagnosis and prognosis of BC. In addition, ANG mediates cleavage within the mature tRNA anticodon loop and is closely related to angiogenesis, depending on its specific cellular localization. In the nucleus, ANG is mainly involved in promoting angiogenesis, whereas in the cytoplasm, ANG acts as a specific ribonuclease that cleaves tRNA [129]. Selitsky et al. [130] found that 5’tRHs are upregulated in chronic viral hepatitis and inhibit tumorigenesis. In addition, the abundance of 5’tRHs is higher in patients with chronic hepatitis B than in those with chronic hepatitis C, and they are down-regulated in patients with HCC. Further studies showed that 5’tRHs levels may be closely related to the expression of ANG, and the differences in the localization and function of ANG in cells may lead to changes in 5’tRHs levels in chronic viral hepatitis and HCC. Li et al. [131] demonstrated that tRF-29-R9J8909NF5JP is upregulated in the serum of patients with GC, which correlates with vascular invasion and TNM staging, suggesting that serum tRF-29-R9J8909NF5JP may be a novel diagnostic and prognostic biomarker for GC.

### tsRNAs and immune response

Tumor immune microenvironment (TIME) refers to the microenvironment surrounding tumor cells, including blood vessels, immune cells, fibroblasts, inflammatory cells, various signaling molecules, and extracellular matrix [132, 133]. However, tumor cells often evade the body’s immune surveillance through a variety of mechanisms, such as inducing endogenous immune checkpoint programmed cell death 1 ligand 1 (PD-L1), enriching immunosuppressive cells, expressing or secreting immunosuppressive factors, and interfering with TIME, thereby avoiding immune recognition and attack [134, 135]. Notably, tsRNAs play a miRNA-like role in the regulation of tumor immunity. As immunotherapy has become widely used as an antitumor therapy, tsRNAs may be an appealing therapeutic tool for targeting immune escape.

In a study of B-cell lymphoma, Maute et al. [136] showed that CU1276, a 22-nt small RNA fragment (5′-TCGATTCCCGGCCAATGCACCA-3′) derived from tRNA-Gly, is down-regulated in lymphoma cells. Mechanistically, CU1276 induces RISC formation together with AGO proteins, playing a miRNA-like role in targeting *RPA1*, a specific gene involved in genome replication. Thus, the downregulation of CU1276 in B-cell lymphoma may confer an obvious growth advantage to malignant B cells. Based on RNA-seq analysis, Chiou et al. [137] revealed that tsRNA levels in the EVs of activated T cells were higher than those of other classes of small RNAs (mainly produced during host defense). These activation-induced tsRNAs in EVs contribute to the inhibition of T-cell activation, which can dynamically regulate T-cell activation signals through enrichment and release within EVs. Moreover, tsRNAs in EVs of activated T cells may mediate intercellular RNA communication, making them highly attractive targets for overcoming tumor immune evasion. In another study, Gao et al. [138] identified 52 tsRNAs using sequencing data of 1,550 plasma samples from patients with NSCLC that were further classified into four subtypes: upstream sequences, 5ʹ-tRF, 3ʹ-tRF, and downstream sequence/1ʹ-tRF. Moreover, the authors demonstrated that 35 tsRNAs (e.g., 5P_tRNA-Thr-CGT-4-1, 3b_tRF-Leu-CCA/CGA, and 5b_tRF-Tyr-GTA) were associated with TIME cell infiltration using the CIBSORT algorithm, and five tsRNAs (e.g., 3P_tRNA-Ser-GCT-6-1, 3P_tRNA-Thr-CGT-4-1, and 3P_tRNA-Arg-TCT-4-1) were correlated with PD-L1. Based on these findings, it has been proposed that tsRNAs play an important role in immune infiltration and may have potential therapeutic value.

### tsRNAs and tumor chemotherapy resistance

Chemotherapy is one of the most important cancer treatment strategies. However, chemoresistance and the adverse side effects of chemotherapeutic agents are the most critical obstacles to chemotherapy [139]. During tumor progression, tumor cells develop chemotherapy resistance through different mechanisms, including genomic changes, signaling pathway regulation, and TME drive [140]. Studies have shown that tsRNAs play critical roles in chemotherapy resistance and are differentially expressed between patients with cancer and normal controls; targeting tsRNAs may provide a new method to treat drug-resistant tumors.

Sun et al. [141] investigated the tsRNAs associated with tamoxifen resistance in hormone receptor-positive BC using high-throughput sequencing technology. The authors established that tRF-16-K8J7K1B is overexpressed in the serum of patients with tamoxifen-resistant BC and can be delivered to sensitive cells via exosomes. Specifically, tRF-16-K8J7K1B highlights tamoxifen resistance by binding to the 3′-UTR of TNF-related apoptosis-inducing ligand, a member of the tumor necrosis factor superfamily, down-regulating apoptosis-related proteins. Thus, tRF-16-K8J7K1B may serve as a prospective predictor for targeting tamoxifen-resistant BC. In addition, a recent study reported that tRF-315, derived from tRNA-Lys, is involved in cisplatin-induced cellular stress responses. Mechanistically, cisplatin upregulates the tumor suppressor genes *GADD45A* and *BAX*, regulating the levels of apoptosis-related proteins and mitochondria-mediated apoptosis in PC cells. However, tRF-315 enhances cisplatin resistance in PC cells by targeting GADD45A and alleviating cisplatin-induced mitochondrial dysfunction. These findings provide new insights into the mechanisms underlying cisplatin resistance in PC and suggest a promising therapeutic target [142]. In addition, Cui et al. [143] revealed that tDR-0009 and tDR-7336 (produced under hypoxic stimulation) were elevated in triple-negative breast cancer (TNBC) and associated with doxorubicin resistance. The authors further found that tDR-0009 and tDR-7336 participated in doxorubicin resistance by regulating STAT3 phosphorylation and maintaining the cancer stem-like cell phenotype [144], which may be potential therapeutic targets in doxorubicin-resistant TNBC.

### tsRNAs and metabolic reprogramming of tumors

Tumor cells face numerous metabolic challenges during tumorigenesis and development, including cell-intrinsic factors such as oncogenes and enzyme mutations, and cell-extrinsic factors such as nutritional changes in the TME, hypoxia, and extracellular acidity [145–147]. Therefore, to overcome various cell-intrinsic and cell-extrinsic factors, adapting to TME with limited nutrient supply and hypoxia, tumor cells undergo many metabolic adaptive transformations, which have been recognized as one of the “hallmarks of cancer” [148, 149]. In recent years, an increasing number of studies have shown that tsRNAs are closely related to several metabolic pathways, thereby providing favorable conditions for the growth and metastasis of tumor cells. Targeting tsRNAs may be an emerging therapeutic strategy in tumor metabolism.

The Warburg effect, also known as aerobic glycolysis, is a well-known manifestation of metabolic dysregulation in cancer and is defined as increased glycolysis and lactate production in cancer cells under aerobic conditions, thereby meeting the nutritional and energy requirements for rapid proliferation and metastasis in tumors [150, 151]. Glucose-6-phosphatase catalytic subunit (G6PC), a key gene in glucose catabolism, plays a significant role in gluconeogenesis and glycogenolysis by affecting the metabolic reprogramming of tumor cells. Zhu et al. [152] demonstrated that tRFLys-CTT-010 is upregulated in TNBC and interacts with G6PC to regulate cellular lactate production and glucose consumption, thereby promoting TNBC cell proliferation and metastasis. This provides new evidence that tsRNAs regulate tumor progression through glucose catabolism and that the tRFLys-CTT-010/G6PC axis may be a potential therapeutic target for TNBC. Furthermore, tsRNAs play essential roles in other metabolic pathways, such as tumor amino acid and lipid metabolism. Based on the analysis of small RNA NGS data, Liu et al. [153] revealed that 5’-tRFCys, derived from the 5’half of cysteine tRNA, is overexpressed during BC progression and metastasis, enhances the stability of target transcripts via promoting the binding of nucleolin to its 5’UTR, and protects target transcripts from exonucleolytic degradation by promoting the oligomerization of nucleolin. The authors further suggest that 5’-tRFCys targets metabolic transcripts, such as platelet-activating factor acetylhydrolase 1 beta 1 (Pafah1b1), which promotes BC cell metastasis via regulating phosphatidylcholine synthesis, and methylenetetrahydrofolate dehydrogenase 1-like (Mthfd1l), a key metabolic enzyme in the folate cycle that affects the production of metabolites (mainly formate and glycine), thereby regulating metabolic pathways for BC progression, assisting BC cell metastasis and leading to poor prognosis. These results suggest that the tRF/Pafah1b1/Mthfd11 axis plays an important role in BC metabolic regulation. Similarly, in a study of CRC, Wang et al. [154] reported that 5’tiRNA-Pro-TGG is elevated in sessile serrated lesions (SSLs), a precancerous lesion of CRC. 5’tiRNA-Pro-TGG targets heparanase 2 (HPSE2) and enhances CRC progression. Based on single-sample gene set enrichment analysis, the authors have suggested that HPSE2 downregulates various metabolic pathways, such as riboflavin, retinol, and cytochrome P450-mediated metabolism. Thus, 5tiRNA-Pro-TGG may be a potential biomarker for the early diagnosis of SSLs.

## Clinical application value of tsRNAs in cancers

### tsRNAs can be served as novel liquid biopsy biomarkers of cancers

Recent studies have shown that tsRNAs are not only involved in tumorigenesis [105, 109, 118, 142, 155–159] (table 2), but also display potential clinical value for early tumor diagnosis and tumor therapy. Advancements in high-throughput technologies and detection platforms have accelerated the exploration of new tumor markers. Single-cell sequencing (SCS) technology identifies variations in the expression of disease-related genes and the distribution of cellular subpopulations. This capability aids in developing potential therapeutic targets for intervention. Therefore, it holds significant relevance in diagnosing and treating various diseases, notably cancers [160, 161]. Liquid biopsy, an emerging non-invasive method, primarily focuses on detecting circulating tumor cells or non-cellular nucleic acid substances in bodily fluids, and has recently gained extensive attention in tumor research [162, 163]. Serving as a complementary approach to histological testing, it holds significance in early tumor screening, aiding treatment selection, and evaluating prognosis. Its minimal invasiveness, rapid assessment, and dynamic monitoring capability render it a valuable tool in these contexts [164]. Increasing studies in recent years have shown that tsRNAs not only exist in tissues, but also widely exist in body fluids (e.g., plasma, serum, urine, cerebrospinal fluid, saliva) of patients with cancer in the form of free or encapsulated in EVs, where they play a role in “cell-cell” communication. Moreover, the expression levels of tsRNAs demonstrate correlations with the progression of different cancers and the clinicopathologic traits of patients. Therefore, tsRNAs can serve as innovative biomarkers in liquid biopsy techniques for diagnosing tumors [42, 165].

Recent investigations have demonstrated promising levels of sensitivity and specificity of tsRNAs observed in tumor tissues and body fluids. The abnormal expression patterns of tsRNAs identified in patients with tumors highlight their potential to act as a new biomarker for tumor diagnosis [166–173] (table 3). In their study, you et al. [166] identified a downregulation in tRF-1:29-Pro-AGG-1-M6 expression in the plasma of patients with LUAD via tRF/tiRNA sequencing. In contrast, an upregulation in tRF-55:76-Tyr-GTA-1-M2 expression was also observed, which was consistent with qRT-PCR results. Moreover, the diagnostic values of these two tRFs were analyzed in LUAD. Specifically, the area under the receiver operating characteristic curve (AUC) were recorded to be 0.882 and 0.896 for tRF-1:29-Pro-AGG-1-M6 and tRF-55:76-Tyr-GTA-1-M2, respectively. Furthermore, the tRF-1:29-Pro-AGG-1-M6 and tRF-55:76-Tyr-GTA-1-M2 expression levels exhibited a close correlation with the expression levels of TNM and carcinoembryonic antigen (CEA), emphasizing their strong diagnostic efficacy. In another study, Wu et al. [168] observed a significant upregulation in the expression level of 5’-tRF-GlyGCC in the plasma of patients with CRC, showcasing an AUC of 0.882 (95% CI: 0.83 to 0.92, *P* < 0.0001). This AUC value (0.882) surpassed that of CEA (0.762) and carbohydrate antigen 199 (CA199, AUC: 0.557), indicating superior diagnostic performance. Furthermore, combining CEA and CA199 with 5’-tRF-GlyGCC significantly enhanced the AUC value to 0.926 (95% CI: 0.87–0.96, *P* < 0.0001). These findings emphasize the potential of 5’-tRF-GlyGCC as a novel biomarker for diagnosing CRC. Additionally, serum tsRNAs showed similar diagnostic value. Jin et al. [169] reported significantly elevated levels of tRF-Pro-AGG-004 and tRF-Leu-CAG-002 in the sera of patients with pancreatic cancer. These levels strongly correlated with postoperative survival and ANG levels, showcasing AUC values of 0.90 and 0.78, respectively. This finding suggests that tRF-Leu-CAG-002 and tRF-Pro-AGG-004 hold promise as novel biomarkers for diagnosing pancreatic cancer. Similarly, the researchers noted a considerable upregulation of tRF-Gln-TTG-006 in the serum mitochondria of patients with HCC. The analysis of ROC curves illustrated that tRF-Gln-TTG-006 shows promise in distinguishing patients with early-stage HCC from normal controls (stage I: sensitivity, 79.0%; specificity, 74.8%). Specifically, the AUC for tRF-Gln-TTG-006 was 0.858 (95% CI: 0.810–0.905) during the differentiation stage I [170]. Zhang et al. [171] noted a high expression of tRF-23-Q99P9P9NDD in the serum of patients with GC. This particular tRF demonstrated effective discrimination between patients with GC and normal controls. Moreover, its expression was significantly linked to lymph node metastasis, TNM stage, neurological/vascular invasion, and the survival outcomes of patients with GC. tRF-23-Q99P9P9NDDA exhibited an AUC value of 0.783 (95% CI: 0.725–0.842), surpassing the AUC values of conventional GC biomarkers like CEA (AUC: 0.715, 95% CI: 0.649–0.780) and CA199 (AUC: 0.614, 95% CI: 0.543–0.686). These findings strongly indicate that when used independently, tRF-23-Q99P9P9NDD shows superior diagnostic potential compared to other established biomarkers for GC. This underscores its ability as a potential biomarker in GC diagnosis. Similarly, tsRNAs contained in exosomes have potential value as biomarkers for early cancer diagnosis. Qian et al. [173] noted that the presence of tRF-20-S998LO9D in exosomes secreted from endometrial carcinoma (EC) was lowered in comparison to levels detected in healthy individuals, exhibiting an AUC value of 0.768. On a mechanistic level, the overexpression of tRF-20-S998LO9D was observed to impede the proliferation and metastasis of EC cells while stimulating apoptosis in these cells. This effect was attributed to the upregulation of SESN2, a set of highly conserved stress-inducible proteins. These findings strongly indicate that exosomal tRF-20-S998LO9D could emerge as an innovative biomarker for the early detection of EC.

**Table 2:** Regulatory mechanism of tsRNAs in tumor progression

Cancer Type	tsRNA Name	tsRNA Type	Expression Level	Target Gene	Downstream Molecules	Cellular Pathway	Biological Functions	Ref.
Gastric cancer	tRF-Val	tRF-3a	↑	EEF1A1	p53, MDM2	MDM2/p53 signaling pathway	Promoted the proliferation and inhibited apoptosis of GC cells	[105]
	tRF-Glu-TTC-027	tRF-1	↓	-	Elk-1, c-Myc, JNK, ERK	MAPK signaling pathway	Suppressed the progression of GC	[155]
	tRF-5026a	5’-tiRNA	↓	-	AKT, PI3K	PI3K/AKT signaling pathway	Inhibited GC cell proliferation	[156]
	tRF-Val-CAC-016	tRF	↓	CACNA1d	Elk-1, c-Myc, JNK, ERK	MAPK signaling pathway	Suppressed the proliferation of GC	[157]
Gallbladder cancer	tRF-3013b	tRF-3b	↓	TPRG1L	c-Myc, p65, p50	NF-κB signaling pathway	Inhibited GBC cell proliferation and induced cell-cycle arrest	[109]
Colorectal cancer	tRF/miR-1280	tRF-3	↓	JAG2	NICD1, Gata1/3	Notch signaling pathway	Suppressed CRC stem cell-like cells and metastasis	[118]
	tRF3008A	tRF-3	↓	FOXK1	c-Jun, c-Myc, CCND1	Wnt signaling pathway	Suppressed the progression and metastasis of CRC	[158]
Prostate cancer	tRF-315	5’-tiRNA	↑	GADD45A	P53, MDM2	p53 signaling pathway	Inhibited PC cell apoptosis and enhance cisplatin resistance	[142]
Pancreatic cancer	tRF-19-PNR8YPJZ	tRF-5	↑	AXIN2	c-Jun, c-Myc	Wnt signaling pathway	Promoted the proliferation and migration of pancreatic cancer	[159]

**Table 3:** Correlation between tsRNAs and clinicopathological features of cancers

Types of cancers	Samples	tsRNAs	Expression	Relationship with clinicopathologic features	AUC	Ref.
Lung adenocarcinoma	Plasma	tRF-1:29-Pro-AGG-1-M6	↓	TNM stage	0.882	[166]
		tRF-55:76-Tyr-GTA-1-M2	↑		0.896	
Non-small cell lung cancer	Exosome	tRF-Ala-AGC-036	↓	TNM stage	0.737	[167]
Colorectal cancer	Plasma	5’-tRF-GlyGCC	↑	Clinical stage	0.882	[168]
Pancreatic cancer	Serum	tRF-Pro-AGG-004	↑	Survival	0.900	[169]
		tRF-Leu-CAG-002	↑		0.780	
Hepatocellular carcinoma	Serum	tRF-Gln-TTG-006	↑	Clinical stage	0.858	[170]
Gastric cancer	Serum	tRF-23-Q99P9P9NDD	↑	Lymphatic metastasis, TNM stage, neurological/vascular invasion, survival	0.783	[171]
		tRF-27-FDXXE6XRK45	↑	TNM stage, lymphatic metastasis, neurological/vascular invasion	0.805	[172]
Endometrial carcinoma	Exosome	tRF-20-S998LO9D	↓	-	0.768	[173]

### tsRNAs can be served as potential targets of cancer therapy

**Fig. 6:**
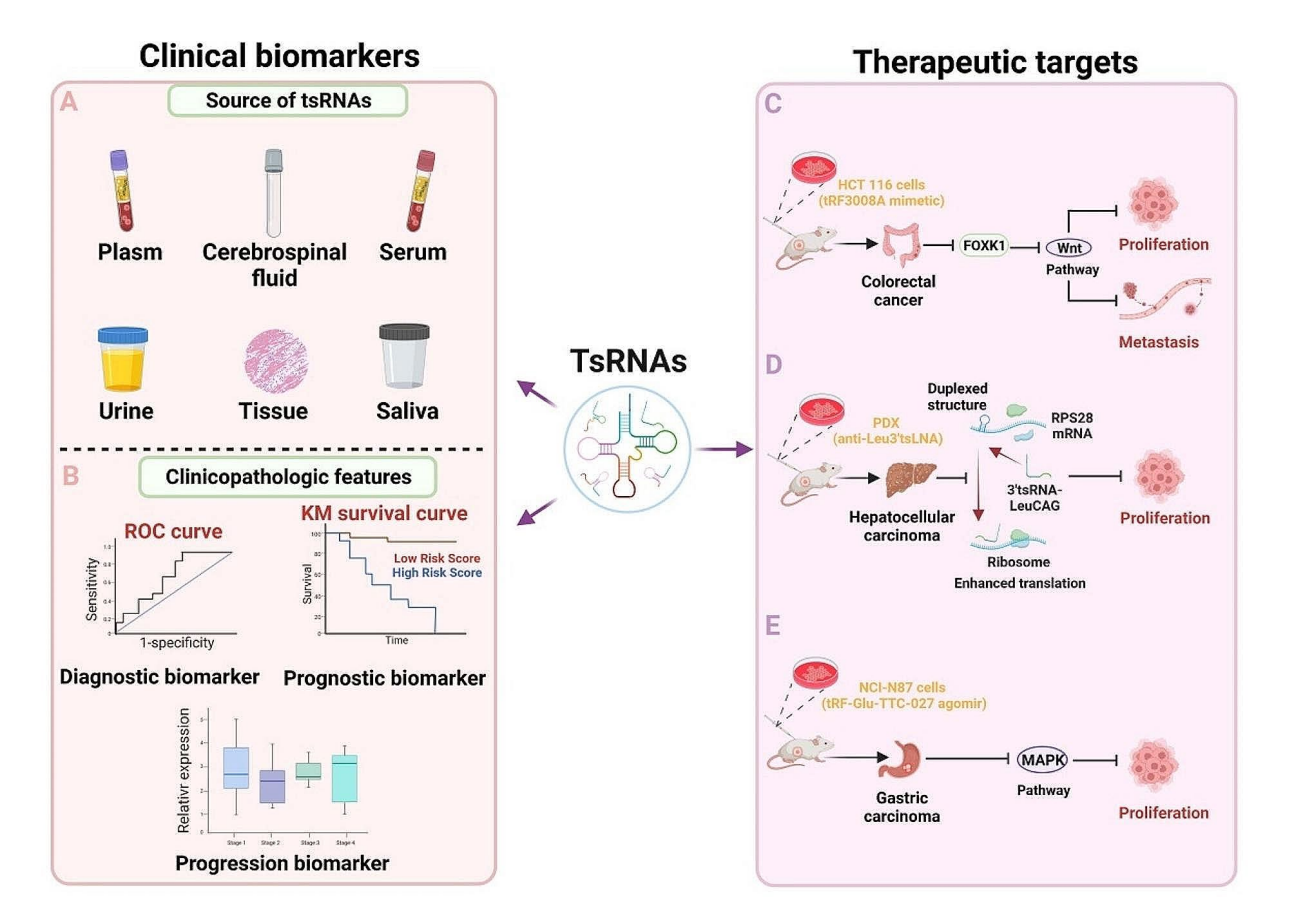
Clinical application of tsRNAs as a cancer diagnostic biomarker and therapeutic target for tumors. (**A**-**B**) tsRNAs can be used as a novel biomarker for cancer diagnosis. tsRNAs are found in human tissues and various body fluids (e.g. plasma, serum, urine, cerebrospinal fluid, and saliva) (**A**); Dysregulation of tsRNAs is closely related to clinicopathological features (such as tumor progression and poor prognosis) (**B**). (**C**-**E**) tsRNAs also have potential as cancer therapeutic targets. In mouse xenotransplantation models, overexpression of tRF3008A inhibited the proliferation and metastasis of CRC cells by inhibiting the FOXK1/Wnt pathway (**C**); anti-leu3’tslna (knockdown 3’tRF-LeuCAG) reduces ribosomal biogenesis by down-regulating RPS28 mRNA translation, thereby inhibiting HCC cell proliferation (**D**); tRF-Glu-TTC-027 agomir (overexpressing tRF-Glu-TTC-027) inhibits GC cell proliferation by inhibiting the MAPK signaling pathway (**E**)

Recent studies indicate that tsRNAs exhibit promise as novel biomarkers for early tumor diagnosis and serve as potential targets for tumor therapy (Fig. 6). For example, Xu et al. [155] revealed a significant downregulation of tRF-Glu-TTC-027 expression in GC through in vitro experiments. It was found that tRF-Glu-TTC-027 was crucial in inhibiting GC progression. In addition, in an in vivo xenograft mouse model, there was a significant decrease in the tumor volume among mice in the tRF-Glu-TTC-027 agomir group (overexpression of tRF-Glu-TTC-027). This outcome was consistent with the results obtained from the in vitro cellular experiments. Additional investigations revealed that tRF-Glu-TTC-027 exerts its inhibitory effect on GC progression by suppressing the MAPK signaling pathway. Moreover, tRF-Glu-TTC-027 emerges as a potential candidate target for GC-targeted therapy. Han et al. [158] evaluated the role of tRF3008A, a tRFRNA derived from tRNA^Val^, in CRC growth and metastasis utilizing a xenograft mouse model. These findings revealed that tRF3008A effectively suppressed CRC cell growth and impeded metastasis in vivo and in vitro. Mice in the tRF3008A mimetics group (tRF3008A overexpression) showed significantly lower tumor growth and lung metastasis than those in the tRF3008A-LNA group (tRF3008 knockdown). Specifically, LNA is a novel chemically modified antisense oligonucleotide (ASO) that regulates gene expression. Mechanistically, tRF3008A demonstrates miRNA-like functions. It binds to AGO, thereby reducing the stability of the oncogenic transcript forkhead box K1 (FOXK1), specifically within CRC cells. This action consequently influences the wnt pathway, inhibiting proliferation and migration in CRC cells. These findings could provide new regulatory factors and therapeutic targets for CRC therapeutic strategies. Similarly, in the HCC-PDX mouse model, Kim et al. [174] observed a significant decrease in tumor volume among the anti-Leu3′tsLNA group (3′tsRNA-LeuCAG knockdown). These findings suggest that the inhibition of 3′tsRNA-LeuCAG could trigger apoptosis in HCC cells. Mechanistically, 3′tsRNA-LeuCAG functions by unwinding the hairpin structure at its target site through base-pairing with the mRNA of ribosomal protein S28 (RPS28). This interaction facilitates the production of ribosomes, consequently elevating the overall translation levels. In contrast, inhibition of 3′tsRNA-LeuCAG results in the downregulation of the translation of RPS28 mRNA. This reduction in translation capacity disrupts ribosome biogenesis, ultimately promoting apoptosis. This discovery implies that targeting 3′tsRNA-LeuCAG could be a viable measure in treating HCC. Similarly, Wang et al. [175] demonstrated that tRF-24-V29K9UV3IU plays a tumor-suppressive role in the development of GC, suppressing the growth and metastasis of these malignant cells. The tumor volume in the LV-tRF-24-inhibitor group (tRF-24-V29K9UV3IU knockdown) was significantly larger than that of normal controls. Additionally, there were more lung metastatic nodules and increased EMT among tumor cells. Mechanistically, tRF-24-V29K9UV3IU acts similarly to miRNA, functioning by downregulating the expression of the chaperone protein G-protein-coupled receptor 78 (GPR78) [176]. This action occurs through its binding to AGO2. Consequently, this inhibition suppresses the growth and migration of GC cells in vitro. These discoveries potentially offer new therapeutic targets for developing innovative treatment strategies aimed at managing GC.

The continuous development and improvement of RNA-related technologies have resulted in the approval of various oligonucleotide drugs and large molecule RNA drugs in the market [177]. These include ASOs, siRNAs, and mRNA vaccines, marking significant progress in the field. RNA therapies involve the use of RNA-based molecules to treat or prevent diseases and hold significance in disease diagnosis and treatment [178]. Yang et al. [179] explored the expression profiles of tsRNAs in experimental traumatic brain injury (TBI) mice. These mice were treated with a Chinese herbal formula, Xuefu Zhuyu Decoction (XFZYD). They screened drug therapy-related tsRNAs and analyzed the functions of these targets by GO and KEGG analyses. This analysis shed light on the potential biological processes involved in drug therapy, indicating that tsRNAs could serve as innovative targets in the XFZYD treatment of TBI. The chemical modification and substantial negative charge as well as the vulnerability of RNAs to nuclease degradation limits their in vivo functionality [180, 181]. However, progress in biomedical materials science, particularly in nanomaterials and nucleic acid aptamers, presents promising solutions to overcome the challenges of delivering oligonucleotide drugs [182]. tsRNAs are abundant and stable in various bodily fluids, showcasing a strong association with the progression and clinicopathological characteristics of different cancer types. They exhibit robust sensitivity and specificity, making them promising candidates for cancer diagnosis and monitoring [36]. Therefore, tsRNAs act as innovative liquid biopsy markers. Utilizing these distinctive tsRNA properties for identifying specific tsRNA aptamers and directing them toward tumor cells holds promise in advancing the development of RNA drugs. This approach aims to enhance the stability and therapeutic effectiveness of these drugs, potentially extending benefits to a broader spectrum of patients with cancer. However, clinical treatments focusing on tsRNAs remain inadequate. Therefore, further in vivo and in vitro studies are needed to elucidate their clinical value as cancer therapeutic targets.

## Discussion and future perspectives

With the rapid development of high-throughput technologies and the establishment of relevant databases, an increasing number of tsRNAs have been discovered and identified. tsRNAs are a class of snRNA fragments produced by pre-tRNAs or mature tRNAs cut at specific sites. They play a “double-edged sword” role (tumor promotion or tumor suppression) during tumorigenesis and development through epigenetic, transcriptional, posttranscriptional, and translational regulation. In addition, with the application of liquid biopsy, a new detection technology, tsRNAs are new liquid biopsy biomarkers and therapeutic targets for tumor diagnosis. Therefore, an in-depth understanding of the roles and mechanisms of tsRNAs in tumorigenesis and development will provide new strategies for early diagnosis and treatment of tumors.

In recent years, with the continuous development and innovation of related research methods and technologies, the application of tsRNAs in tumor diagnosis has begun to emerge. (1) tsRNAs may serve as pathological diagnostic markers for patients with tumors. In recent years, researchers have used fluorescence in situ hybridization (FISH) kits to detect the abnormal expression of tsRNAs in tumor tissues for early diagnosis, therapeutic monitoring, and prognostic evaluation [74]. It is safe, rapid, and highly sensitive, and has been widely used for molecular-level gene detection and auxiliary diagnosis of tumors [183]. (2) tsRNAs may be used as liquid biopsy markers in patients with tumors. tsRNAs are highly abundant and stable in a series of body fluids and are closely associated with the clinicopathological characteristics of patients, suggesting that tsRNAs may be novel liquid biopsy biomarkers for tumor diagnosis. In addition, human plasma contains various exogenous RNAs derived from bacteria and fungi (including many tRNA fragments), which mediate communication between microorganisms and host cells, and host-pathogen interactions through the release of various RNAs wrapped in EVs [27, 184]. For example, EVs derived from Helicobacter pylori can be absorbed by gastric epithelial cells and enhance the carcinogenic potential of the bacteria, which is an adverse event in the occurrence of GC [185]. These findings provide new insights into the mechanism of tsRNA-mediated intratumoral microbial regulation of tumorigenesis and tumor development. (3) Deep-sequencing technology can assist with tsRNA detection. Some researchers have proposed that the development of artificial intelligence (AI), machine learning, and microfluidic biochip technology will enhance our ability to identify tsRNAs involved in tumor diagnosis [186, 187]. For example, artificial neural networks are used to integrate multiple layers of tsRNA information (e.g., biogenesis, site-specific RNA modifications, and expression levels), which will help identify potential biomarkers for tumor diagnosis [9, 188]. Moreover, the use of high-throughput sequencing combined with novel microfluidic biochips enabled rapid, simple, and highly sensitive detection of tsRNA diagnostic features in esophageal squamous cell carcinoma [186]. Furthermore, the application of tsRNAs in combination with biomedical materials opens new avenues for cancer treatment [189].

Although a growing number of studies have shown that tsRNAs play important roles in tumorigenesis and development, the study of tsRNAs still faces many challenges and limitations: (1) Many unknown tsRNAs still need to be uncovered and identified. Although several databases have been established for potential functional prediction and mechanistic studies of tsRNAs, the number of tsRNAs contained in these databases is still limited, and many tsRNAs newly discovered by high-throughput sequencing have not been uncovered and identified. Therefore, it is necessary to establish a high-coverage database and combine it with new sequencing technologies (e.g., SCS and AI) to comprehensively analyze tumor-specific tsRNAs. (2) tsRNAs lack a standardized naming system. tsRNAs are fragments generated by cleavage of tRNAs at specific sites, not random degradation. However, tsRNA production is affected by various cellular stresses. In addition, numerous RNA modifications are present in tRNAs, and the potential relationship between them is currently unknown. Based on the biogenesis and classification of tsRNAs, different tsRNA nomenclatures have emerged, which leads to the same or similar tsRNAs having completely different names in different studies. Therefore, standardization of tsRNA nomenclature is crucial for further research. (3) tsRNAs lack mature research systems. An increasing number of studies have found that tsRNAs can regulate the expression of their corresponding target molecules at the epigenetic, transcriptional, posttranscriptional, and translational levels, thereby exerting biological functions. However, the methods to investigate the regulatory functions of tsRNAs are not yet mature. siRNA and ASO are common approaches for oligonucleotide therapy research, but the application of these technologies with tsRNAs often faces challenges [190]. For example, tsRNAs have the same sequences as pre-tRNAs and some homologous tsRNAs, and thus it will produce off-target effects, and ASO is easily degraded. CRISPR/Cas9-based gene editing modifies endogenous genes of various species and cell types, with high efficiency and low off-target effects [191–193]. For example, Balatti et al. [52] generated a stable knocked-out (KO) cell model for ts-101 and ts-46 in HEK-293 cells using CRISPR/Cas9 technology and found that tsRNAs are involved in the epigenetic control of gene expression and are associated with the activation of oncogenes and the inactivation of tumor suppressors. tsRNAs may therefore provide new strategies for diagnosis and targeted therapy of tumors. CRISPR/Cas9 technology has been widely used in tRNA-associated research, although it has rarely been used in tsRNA-associated research [194, 195]. (4) Studies on tsRNAs as tumor therapeutic targets are insufficient. Although several studies have analyzed tumor progression after targeting the corresponding tsRNAs with inhibitors, most remain at the animal experimental stage and lack human experimental data. Therefore, further studies are required to determine whether tsRNAs can be translated from basic research to clinical applications.

Although our current understanding of tsRNAs is only the tip of the iceberg, with continuous innovations in technology and research methods, these bottlenecks will eventually be resolved. As an emerging field, tsRNAs have broad application prospects in tumor research, providing new directions and possibilities for early diagnosis and treatment of tumors.

## Data Availability

No datasets were generated or analysed during the current study.

## Abbreviations

tRNA: transfer RNA
tRNA-derived small RNAs: tsRNAs
ncRNAs: Non-coding RNAs
lncRNAs: Long non-coding RNAs
sncRNAs: Small non-coding RNAs
miRNAs: microRNAs
siRNAs: Small interfering RNAs
piRNAs: PIWI-interacting RNAs
tRFs: tRNA-derived fragments
tiRNAs: tRNA halves
EVs: Extracellular vesicles
NSCLC: Non-small cell lung cancer
GC: Gastric cancer
PC: Prostate cancer
HCC: Hepatocellular carcinoma
CRC: Colorectal cancer
TNBC: Riple-negative breast cancer
GBC: Gallbladder cancer
iPSCs: Induced pluripotent stem cells
ESCs: Embryonic stem cells
Pol III: RNA polymerase III
m5C: 5-methylcytosine
m7G: N7-methylguanosine
ChIP: Chromatin immunoprecipitation
RISC: RNA-induced silencing complex
YB-1: Y-box-binding protein 1
PABPC1: Poly(A)-binding protein cytoplasmic
GADD45G: Growth arrest DNA damage-inducible gene 45 gamma
FGL1: Fibrinogen-like protein-1
AURKA: Aurora kinase A
EMT: Epithelial-mesenchymal transition
TME: Tumor microenvironment
TIME: Tumor immune microenvironment
PD-L1: Programmed cell death 1 ligand 1
HPSE2: Heparanase 2
G6PC: Glucose-6-phosphatase catalytic subunit
FOXK1: Forkhead box K1
AXIN2: Axis inhibition protein 2
RPS28: Ribosomal protein S28
GPR78: G-protein-coupled receptor 78
SSLs: Sessile serrated lesions
FISH: Fluorescence in situ hybridization
KO: Knocked-out
SCS: Single-cell sequencing
AI: Artificial intelligence
ASO: Antisense oligonucleotide
